# Improving diffusion-weighted imaging of post-mortem human brains: SSFP at 7 T

**DOI:** 10.1016/j.neuroimage.2014.08.014

**Published:** 2014-11-15

**Authors:** Sean Foxley, Saad Jbabdi, Stuart Clare, Wilfred Lam, Olaf Ansorge, Gwenaelle Douaud, Karla Miller

**Affiliations:** a FMRIB Centre, Nuffield Department of Clinical Neurosciences, University of Oxford, Oxford, UK; b Division of Clinical Neurology, University of Oxford, Oxford, UK

**Keywords:** Post-mortem human brain, Diffusion-weighted steady-state free precession, 7 T, Tractography, Diffusion tensor imaging, MRI

## Abstract

Post-mortem diffusion imaging of whole, human brains has potential to provide data for validation or high-resolution anatomical investigations. Previous work has demonstrated improvements in data acquired with diffusion-weighted steady-state free precession (DW-SSFP) compared with conventional diffusion-weighted spin echo at 3 T. This is due to the ability of DW-SSFP to overcome signal-to-noise and diffusion contrast losses brought about by tissue fixation related decreases in T_2_ and ADC. In this work, data of four post-mortem human brains were acquired at 3 T and 7 T, using DW-SSFP with similar effective b-values (b_eff_ ~ 5150 s/mm^2^) for inter-field strength comparisons; in addition, DW-SSFP data were acquired at 7 T with higher b_eff_ (~ 8550 s/mm^2^) for intra-field strength comparisons. Results demonstrate that both datasets acquired at 7 T had higher SNR and diffusion contrast than data acquired at 3 T, and data acquired at higher b_eff_ had improved diffusion contrast than at lower b_eff_ at 7 T. These results translate to improved estimates of secondary fiber orientations leading to higher fidelity tractography results compared with data acquired at 3 T. Specifically, tractography streamlines of cortical projections originating from the corpus callosum, corticospinal tract, and superior longitudinal fasciculus were more successful at crossing the centrum semiovale and projected closer to the cortex. Results suggest that DW-SSFP at 7 T is a preferential method for acquiring diffusion-weighted data of post-mortem human brain, specifically where the primary region of interest involves crossing white matter tracts.

## Highlights

• Diffusion weighted SSFP imaging of post-mortem human brain at 7 T is compared to 3 T.
• Both SNR and diffusion contrast are improved at 7 T.
• 7 T data show improved crossing fiber estimates and accompanying tractography.
• Including B_1_ map in 7 T data analysis brings diffusion metrics in line with 3 T data.

## Introduction

Diffusion tensor imaging (DTI) is an increasingly popular magnetic resonance imaging (MRI) technique for probing the microscopic architecture of white matter in the brain. The predominant confounding factor affecting tractography reconstruction from in vivo DTI data is the experimental time-scale. High-resolution imaging (with voxel sizes smaller than ~ 2 mm) generally requires long scan times as well as acquisition of a large number of diffusion-weighted (DW) image volumes to overcome the associated signal-to-noise ratio (SNR) losses. This puts an immediate strain on the subject/patient to remain still for unreasonable durations.

An alternate method for validating small tracts or fine tract features is to image post-mortem human brain (D'Arceuil and de Crespigny, 2007, D'Arceuil et al., 2008, Kolasinski et al., 2012, McNab et al., 2009, Miller et al., 2011, Miller et al., 2012). Post-mortem tissue provides the advantage of having no time restrictions, thereby allowing for the long scan durations necessary to achieve high resolutions that are unfeasible in vivo. DTI data of post-mortem rat brain has been acquired with isotropic voxel sizes as small as 43 μm (Jiang and Johnson, 2010), albeit requiring gradient strengths of up 2000 mT/m. This suggests that the prohibitive factors required to push the limits of post-mortem DTI are not inherent signal limitations, but rather experimentally dependent technical difficulties related to available scan time and state-of the-art hardware.

By performing tractography on DTI data collected in post-mortem human brain, we are provided with a non-invasive method for validating tracts that could otherwise only be localized using techniques that are destructive to the tissue, including dissection, histological fiber visualization, and tracer methods (Alarcon et al., 2013, Dyrby et al., 2007, Gutman et al., 2013, Leergaard et al., 2010, Ruest et al., 2011, Schmahmann et al., 2007). Tissue preservation is a powerful benefit, allowing for subsequent investigative techniques on otherwise undamaged tissue samples.

There are several reported technical challenges that require attention for post-mortem imaging to become optimal: the post-mortem interval (PMI, the time between death and commencement of tissue fixation) (D'Arceuil and de Crespigny, 2007, Miller et al., 2011, Shepherd et al., 2009), the scan interval (SI, the duration the sample remains in fixative) (Dawe et al., 2009), and the time-dependent consequences both of these have on tissue properties and MR measurements. For example, the fractional anisotropy (FA) and apparent diffusion coefficient (ADC) have both been reported to decrease with increased PMI (D'Arceuil and de Crespigny, 2007). Tissue samples stored in fixative prior to imaging have shown decreased tissue ADC, proton density, and T_2_/T_2_^⁎^ compared with in vivo experiments (D'Arceuil and de Crespigny, 2007, Pfefferbaum et al., 2004, Sun et al., 2003, Sun et al., 2005).

Fixation related alterations of MR measurements have unfavorable effects on DW MR experiments in post-mortem brain tissue: they exacerbate already competing demands between SNR and diffusion contrast, producing a tradeoff between TE and b-value. On the one hand, the TE can be decreased to improve SNR, however achievable b-values become limited to those comparable to in vivo studies due to the decreased ADC; on the other, increased b-values can be achieved, but require longer TEs and subsequent decreased SNR due to shortened T_2_.

DTI studies of post-mortem brain are generally conducted using some variation of a DW spin-echo (DW-SE) protocol (Englund et al., 2004, Larsson et al., 2004, Stejskal and Tanner, 1965). While DW-SE is a robust and efficient approach to in-vivo DW imaging, it is not necessarily the ideal method for post-mortem brain. We have demonstrated that a DW adaptation of the steady-state free precession pulse sequence (DW-SSFP) (McNab and Miller, 2010, Merboldt et al., 1989) out-performs DW-SE at 3 T, both in terms of tensor fits (McNab et al., 2009) and tractography (Miller et al., 2012). These results are driven by higher SNR efficiency in DW-SSFP since data are collected with much shorter TEs, achieving high b-values without severe T_2_ signal loss. In fact, the primary reason why DW-SSFP is not used in-vivo is due to intense motion sensitivity (McNab and Miller, 2010, Merboldt et al., 1989), which is clearly not a problem for post-mortem imaging. Nevertheless, previous efforts to extract crossing fiber estimates from DW-SSFP data at 3 T met with mixed success (Miller et al., 2012), indicating that although sub-millimeter resolution was feasible, CNR is still inferior to in-vivo imaging.

In this work, we present improvements in DW-SSFP imaging of post-mortem brains that reflect several advances in hardware, most prominently the use of a 7-Tesla scanner. It is presumed that because of higher field (7 T vs. 3 T), more receiver channels (32 ch vs. 12 ch), and increased achievable gradient strength (70 mT/m vs. 40 mT/m) on this system, we could realize increases in contrast-to-noise ratio (CNR) that would improve secondary fiber estimates compared to previous work. We acquired DTI data using DW-SSFP in four post-mortem human brains at 3 T and 7 T with matched effective b-value (b_eff_ ≈ 5150 s/mm^2^), as well as increased b_eff_ (8500 s/mm^2^) at 7 T. Simple imaging metrics were computed to assess data quality and estimates of diffusion direction. Data at 7 T were found to be superior in terms of SNR and diffusion contrast. Moreover, data acquired with a higher b_eff_ have more precise estimates of secondary fiber populations than lower b_eff_, confirming that 7 T scanning supports the use of b-values that are more equivalent to those used in vivo. We then investigated whether these gains improved tractography results through regions where tracts are known to interdigitate in the centrum semiovale (CS): the corpus callosum (CC), corticospinal tract and other related motor cortex projections (CST), and superior longitudinal fasciculus (SLF). 7 T data produced superior tractography results where dependence on secondary fiber populations through the CS is critical. Finally, an analysis of the effects that characteristic B_1_ inhomogeneities at 7 T have on fractional anisotropy and mean diffusivity were investigated to further assess data improvements at high field due to the addition of a B_1_ map in the processing pipeline.

## Methods

### Tissue preparation

Data were acquired of post-mortem human brains (n = 4) diagnosed with Alzheimer's disease (n = 2), Parkinson's disease (n = 1), and motor neuron disease (n = 1). Brains were extracted from the skull within 72 h after death and fixed in 10% PBS buffered formalin (4% formaldehyde) for at least 2 months prior to scanning. Brains were removed from formalin and placed in plastic bags filled with a perfluoropolyether liquid (Fomblin LC08, Solvay Solexis Inc.).^1^ Bagged samples were placed on a rocker table and gently agitated for a minimum of 6 h to remove air bubbles from the cortical sulci and ventricles. Because data were acquired on two different scanners, this non-rigid packing procedure generally resulted in minor non-linear deformations of the brain; this did not prevent broad comparisons between data acquired at different field strengths but did prevent the possibility of reliable voxel-wise comparisons.

### MRI scanning protocol

#### DW-SSFP

All samples were imaged with both a 3 T Siemens Trio and a Siemens 7 T scanner. In both cases, DW data were acquired using a 3D DW-SSFP pulse sequence. Unlike our previous work, acquisitions at both field strengths utilized a single-line readout rather than the previously implemented segmented-EPI readout (Miller et al., 2012). The higher field strength produced a sufficient decrease in T_2_ (T_2_^⁎^) to render a single-line readout approach more SNR efficient. This was done with a severe cost in extended scan duration, however unrestricted scan times is one of the predominant advantages of post-mortem imaging. All specific DW data acquisition protocol parameters are reported in Table 1a.

**Table 1 MR imaging protocol parameters.** 

a. DW-SSFP	3 T q = 300 cm^− 1^	7 T q = 300 cm^− 1^	7 T q = 400 cm^− 1^
Coil	12 channel	32 channel	
b_eff_ (s/mm^2^)	5175	5150	8550
TE/TR (ms)	26/35	21/30	25/34
Resolution (mm)	1.0 × 1.0 × 1.0	1.0 × 1.0 × 1.0	1.0 × 1.0 × 1.0
Flip angle (°)	35	30	30
Averages	2	2	2
Diffusion grad duration (ms)	19	13	17
Diffusion gradient strength (mT/m)	38	56	70
Number of directions	52	49	49
Bandwidth (Hz/pixel)	159	80	80
Duration 1 volume (min:s)	10:48	11:27	11:27
Total duration (h:min:s)	20:09:36	20:13:42	20:13:42


b. Structural 3D-TRUFI	3 T	7 T
TE/TR (ms)	26/35	3.79/7.58
Resolution (mm)	0.5 × 0.5 × 0.5	0.35 × 0.35 × 0.4
Flip angle (°)	37	35
Averages	8–12 without phase cycling 8–12 with 180° phase cycling	8–12 without phase cycling 8–12 with 180° phase cycling
Bandwidth (Hz/pixel)	303	296

c. B_1_-map 3D-AFI	3 T	7 T

TE/TR1/TR2 (ms)	–	2.35/6.0/30.0
Resolution (mm)	–	2.0 × 2.0 × 2.0
Flip angle (°)	–	60
Averages	–	2
Bandwidth (Hz/pixel)	–	260

d. T_1_-map Turbo spin-echo	3 T	7 T

TE/TR (ms)	12.0/1000	12.0/1000
Resolution (mm)	1.4 × 1.4 × 1.4	1.0 × 1.0 × 1.4
Flip angle (°)	180	180
Averages	1	1
Bandwidth (Hz/pixel)	200	200
TIRs (ms)	30, 60, 120, 240, 480, 900	32, 64, 125, 250, 500, 850

e. T_2_-map Turbo spin-echo	3 T	7 T

TEs (ms)	14, 28, 42, 56, 71	14, 28, 42, 56, 70, 84
TR (ms)	1000	1000
Resolution (mm)	1.4 × 1.4 × 1.4	1.0 × 1.0 × 1.4
Flip angle (°)	180	180
Averages	1	1
Bandwidth (Hz/pixel)	159	130

Unlike the classical Stejskal–Tanner DW-SE pulse sequence in which the diffusion coefficient is simply related to the exponential signal decay, DW-SSFP relies on a more complex signal model (Buxton, 1993). By this formalism, the DW signal is dependent upon the voxel-by-voxel T_1_, T_2_, and flip angle (B_1_). To properly compute the voxel-wise ADC, it was, therefore, necessary to acquire maps of these three parameters (McNab et al., 2009).

Previous reports of DW-SSFP for post-mortem imaging used segmented EPI acquisitions, for example acquiring 10 lines of k-space per TR, producing a volume in just over 1 min of scan time (Miller et al., 2012). While the EPI approach may appear appealing, there are several advantages to acquiring just a single line in the same time the previous protocols dedicated to an EPI segment, despite the fact that this modified approach increases scan time (e.g. in this study a volume every ~ 11 min). First, the reduced bandwidth of our single-line readout corresponds to a lower gradient amplitude, reducing heating and thus the minimum achievable TR (here, reduction from 42 ms to 34 ms means that the fraction of TR dedicated to signal acquisition increases from 26% to 37%). Second, by abandoning segmented EPI there is no need to dedicate any time to phase correction scans, nor is there any EPI ghosting. Third, even under SNR efficiency, longer volume scan times reduce noise rectification in magnitude data (Gudbjartsson and Patz, 1995), which is particularly important in the low SNR regime of diffusion-weighted data. The first two effects increase SNR efficiency in our single-line implementation, while the third reduces the effective noise across a series of image volumes.

Because diffusion weighting with SSFP is dependent on the prescribed acquisition flip angle and TR, as well as tissue T_1_ and T_2_, a traditional b-value is not well defined. However, we can estimate an effective b-value (b_eff_) that reflects the diffusion contrast given the more appropriately defined q-value (Miller et al., 2012). Therefore, b_eff_ was estimated using the following average values computed from white matter masked measurements: ADC = 0.8 × 10^− 4^ mm^2^/s, and T_1_/T_2_ = 520/55 ms and 595/37 ms at 3 T and 7 T, respectively.

Without the inclusion of the diffusion-weighting gradient in the DW-SSFP pulse sequence, the gradients become balanced and subsequent data are susceptible to banding artifacts (Zur et al., 1988). Because of this, scans were acquired with a slight diffusion gradient applied to serve as a spoiler. These data are hereafter referred to as b_eff_ = 0 s/mm^2^, but did in fact have small b-values (b_eff_ = 6.4 and 8.0 s/mm^2^ at 3 T and 7 T, respectively).

#### Structural

As previously reported (Miller et al., 2011), because of the convergence of T_1_ in gray matter and white matter in post-mortem tissue, conventional T_1_-weighted structural protocols do not produce usable gray/white matter contrast. A 3D balanced SSFP pulse sequence was implemented instead, producing high gray/white matter contrast, albeit inverted compared with conventional T_1_ weighted structural acquisitions (gray matter has high relative signal; white matter, low). Details of the acquisition parameters are reported in Table 1b. Balanced SSFP data are sensitive to B_0_ inhomogeneities, resulting in the aforementioned banding artifacts (stripes of low signal). To account for this, data were acquired in “phase-cycled” pairs in which the regions of low signal in one dataset have high signal in the other. An average structural dataset was produced from the RMS of all structural scans, effectively removing banding artifacts in regions where susceptibility-related gradients were relatively small (i.e. slowly spatially varying banding artifacts).

### Data analysis

All data were processed and analyzed using the FMRIB software library (FSL) (Smith et al., 2004, Woolrich et al., 2009) and Matlab (The MathWorks Inc., Natick, MA, 2000). Individual image volumes were co-registered to account for eddy current and B_0_ drift (Miller et al., 2011) using affine registration (FLIRT: FMRIB linear registration tool) (Jenkinson and Smith, 2001). Processed diffusion data were co-registered with the respective structural scan by a second affine transformation. Finally, 3 T and 7 T structural data for each sample were co-registered by a third affine transformation. By applying this series of co-registrations, inter-sample comparisons were possible (note that all linear transforms were concatenated and a single transform was applied to the data to avoid multiple resamplings). All comparative analyses were done solely over white matter; white matter masks were produced from mean diffusivity maps using FAST (FMRIB automated segmentation tool) (Zhang et al., 2001).

SNR was computed to assess potential gains of high field strength. The average signal intensity over the entire white matter masked data was measured in all 3 T and 7 T b_eff_ = 0 s/mm^2^ datasets, and the SNR (average ± standard deviation) of all pooled 3 T and 7 T data was computed.

Diffusion contrast was estimated from the angular uncertainty in both the primary and secondary fiber population estimates produced using a modified version of BEDPOST (Behrens et al., 2007, McNab et al., 2009). Normalized histograms of these uncertainty estimates were produced, and the mode of the distributions was calculated. Uncertainty in direction estimates is a primary determinant of how well tractography works and is driven primarily by diffusion contrast, so is used here as a proxy for the robustness of contrast (Jbabdi et al., 2012, Sotiropoulos et al., 2013). Specifically, the uncertainty is calculated using a measure of dispersion in the orientations of samples from the posterior distribution of fiber orientations. The dispersion is a scalar value defined as 1 − Λ, where Λ is the largest eigenvalue of the average dyadic tensor of all posterior sample orientations. This leads to a scalar between 0 and 1 (i.e. low and high dispersion/uncertainty).

The modification in BEDPOST was made to include the DW-SSFP signal equation and thereby account for associated diffusion contrast differences (McNab and Miller, 2008). Of particular importance in the modified version is the inclusion of T_1_, T_2_, and B_1_ information to allow for accurate voxel-wise estimates of diffusion coefficients. An analogous modified version of DTIFIT was used for DTI measurements.

**Fig. 1:**
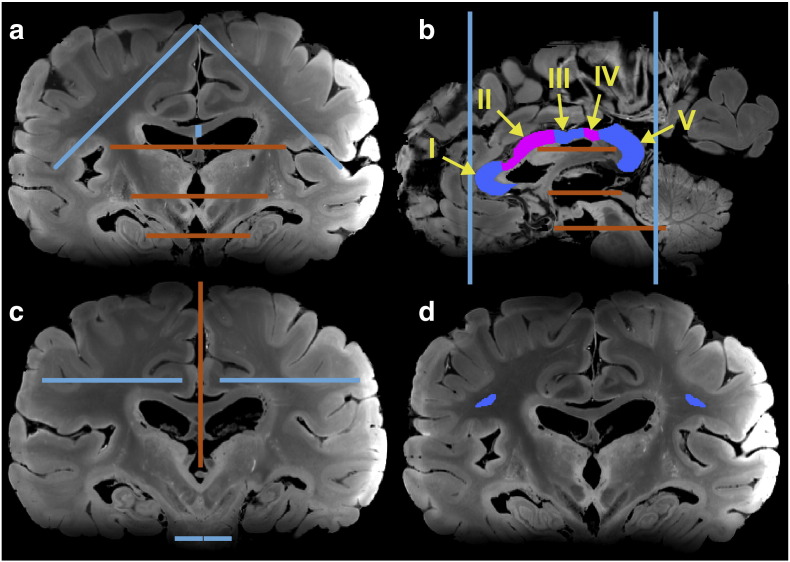
(a) Inclusion (blue) and exclusion masks (orange) for CC. Inclusion masks extended A/P across the whole brain, where as exclusion masks only extended the width of the CST. (b) CC seed (I–V), inclusion (blue) and exclusion (orange) masks. Inclusion masks extended L/R across the whole brain where as exclusion masks only extended the width of the CST. (c) CST inclusion (blue) and midline exclusion (orange) masks. The inferior inclusion mask was used as the seed. (d) Simple seed mask for the SLF.

Tractography was performed specifically to identify effects of secondary fiber population estimates. Seed masks (Fig. 1) were defined in regions where tracts of interest are conventionally identified (e.g. seeding the midline of the CC (Hofer and Frahm, 2006)); however, inclusion masks were defined to exploit the presence (or lack) of interdigitating fibers through the CS. All inclusion masks for a particular tract were liberally defined to include targets a respective tract was expected to reach, but on the distal side of the CS from the respective seed mask. Reconstructed tracts thus included only streamlines that passed from the seed mask and reached the inclusion mask via the CS. The extent to which this occurs is an indication of the prevalence and coherence of secondary fiber populations.

The aforementioned modified version of BEDPOST was used to estimate the probability distribution for the fiber orientation in each voxel. Tractography was performed on the modified BEDPOST results using PROBTRACKX2 (Behrens et al., 2003). Parameters included a step-length of 0.2, 5000 streamlines, and no curvature threshold.

Maximum intensity projection images (MIPs) were produced for each tract along a specified direction to improve the visualization of general tractography results. All MIPs were overlain on an appropriate slice of the structural data acquired at 7 T with the understanding that the selected slice merely represented a generalized view of the tract location.

## Results

### SNR

SNR (described above) of all pooled 3 T and 7 T was computed to assess the potential for achievable gains in performing post-mortem DW imaging at high field strength. The measured SNR at 7 T (66 ± 18) was significantly larger than that at 3 T (16 ± 7, *p* = 0.006 by two-tailed Student's t-test). There are several factors that contribute to differences in SNR across the two scanner setups. Assuming a linear dependence of SNR on field strength, we would expect a gain of 2.33 at 7 T compared with 3 T. Increased signal averaging due to the increased number of channels from 12 to 32 at 3 T and 7 T, respectively, would produce a further gain of 1.6 at 7 T (although further gains might be expected due to smaller coil elements). The DW-SSFP signal model predicts a decrease in SNR at 7 T of 0.74 times the SNR at 3 T due to changes in T_1_ and T_2_. Finally, the decrease in receiver bandwidth from 159 Hz/pixel to 80 Hz/pixel at 3 T and 7 T, respectively, provides a further gain of ~ 1.4 at 7 T. Therefore, the expected change in SNR at 7 T (the product of the aforementioned adjustments) is predicted to be approximately 3.9 times the SNR at 3 T, which is largely in agreement with the ~ 4.1 factor gain observed.

**Fig. 2:**
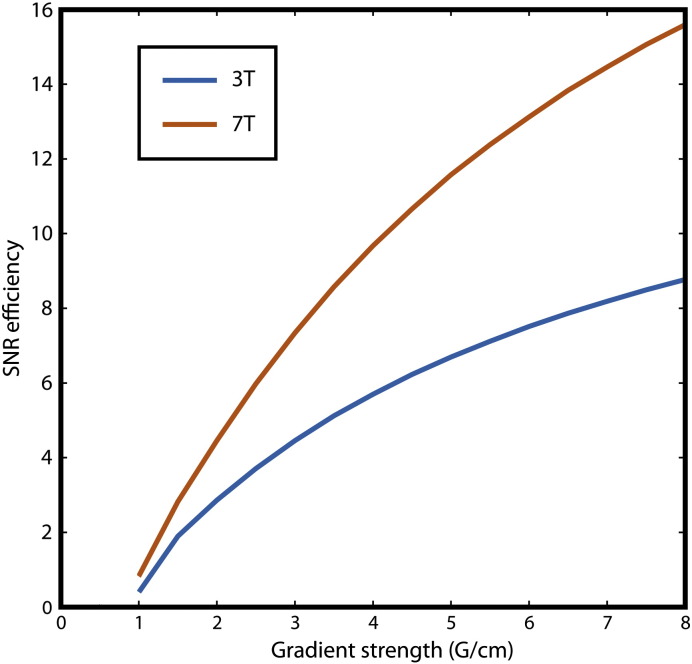
Optimized SNR efficiency at a range of gradient strengths for identical 3 T (blue) and 7 T (red) systems. T_1_/T_2_, and ADC values required for the DW-SSFP signal model used to computer SNR efficiency were 520/55 ms, 595/37 ms, and 1.5 × 10^− 4^ mm^2^/s at 3 T and 7 T, respectively.

In addition to SNR, the SNR efficiency was calculated for 3 T and 7 T over a range of gradient strengths. SNR efficiency was computed as the product of the field strength dependent SNR optimized DW-SSFP signal (using aforementioned field dependent T_1_, T_2_, and ADC) and the square root of the fraction of the TR period dedicated to signal acquisition. The model assumes that the 3 T and 7 T systems are identical except for field strength. Fig. 2 demonstrates that the effect of increased polarization due to higher field strength outweighs the field strength dependent changes in T_1_ and T_2_, indicating that the 7 T system should out-perform a similar 3 T system.

### CNR and secondary fiber estimates

#### Inter-field strength comparison (3 T vs. 7 T, b_eff_ ≈ 5150 s/mm^− 2^)

**Fig. 3:**
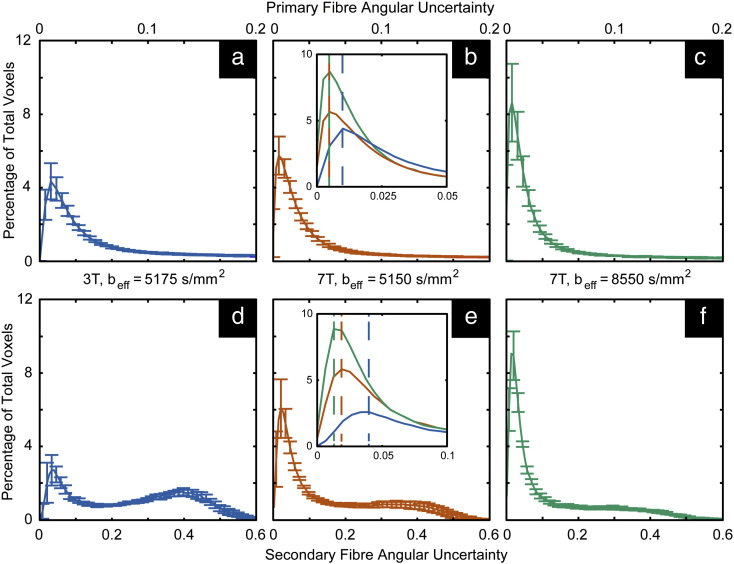
Normalized histograms of angular uncertainty estimations pooled over all white matter masked data (± standard error). Estimates are for (a–c) the primary diffusion direction and (d–f) the secondary fiber diffusion direction. The insets in b and e are magnifications of the modes of the respective three histograms allowing for a clearer visualization between protocol variations in the distributions.

Because diffusion contrast is direction dependent, it is not trivial to compute the CNR from diffusion data. We instead use the computed angular uncertainty of the primary and secondary fiber estimates (from the aforementioned modified version of BEDPOST) as a proxy for CNR, where high CNR would in general result in low uncertainty. Normalized histograms were generated of the voxel-wise angular uncertainty in pooled 3 T and 7 T data acquired with the same b_eff_ value. These provide a method of direct comparison of diffusion contrast between results at 3 T and 7 T. These estimates are shown in Figs. 3a,b and d,e for primary and secondary fibers, respectively. All primary fiber distributions were found to be statistically significantly different from one another by a non-parametric, two-sample Kolmogorov–Smirnov (K–S) test (*p* < 0.02); the same was found for the distributions of secondary fibers by the same K–S test (*p* ≪ 0.001). In both the primary and secondary fiber angular uncertainty estimates, the distribution mode is at a higher uncertainty at 3 T than at 7 T, and the distribution is broader (see insets in Figs. 3b and e, which show magnifications of the distribution). This indicates that 7 T data have a greater relative proportion of voxels with more accurate estimates of both primary and secondary fiber diffusion directions, and that the range of estimates is smaller (i.e. more precise) at 7 T than at 3 T, given the same diffusion weighting. Additionally, we see a hump in the secondary fiber histogram at 3 T at very high angular uncertainty (~ 0.4) that is not present at 7 T, indicating a large proportion of voxels with very high angular uncertainty. These results suggest that the diffusion CNR at 7 T is higher than that at 3 T.

#### Intra-field strength comparison (b_eff_ = 5150 s/mm^− 2^ vs. 8550 s/mm^− 2^, 7 T)

Our 7 T scanner has a higher achievable maximum gradient strength than our 3 T system (70 and 38 mT/m, respectively), making it useful to determine potential gains of larger b_eff_. Figs. 3b,c and e,f demonstrate an analogous comparison of histograms as those described above (Inter-field strength comparison (3T vs. 7T, beff≈5150s/mm−2)), but for intra-field strength variations in b_eff._. The mode of the data acquired with lower b_eff_ has higher uncertainty and a broader distribution than data acquired with higher b_eff_. This suggests that higher b_eff_ produces more accurate estimates of both the primary and secondary fiber populations' diffusion directions and in a larger relative number of voxels at 7 T.

Histograms of secondary fiber estimates described in Fig. 3 are normalized by the number of voxels, allowing for the above comparison of relative percentages as a function of angular uncertainty. Another method of measuring data quality is to count the number of voxels that have secondary fiber estimates. Because of the mechanics of the automatic relevance determination (ARD) prior in BEDPOSTX, uncertainty in fiber orientation will be related to the number of secondary fibers. The volume fraction parameter is driven to zero when there is high uncertainty in the corresponding direction (i.e. little explanatory power). However, this is just a preliminary indicator of data quality. Subsequent tractography is required to confirm that secondary fiber population estimates are sensible. The actual number of voxels (mean percentage of the white matter mask ± standard deviation) in which estimates are produced were 21.5 ± 2.2, 30.2 ± 7.6, and 24.9 ± 3.6% for diffusion protocols b_eff_ = 5175 s/mm^2^ at 3 T, b_eff_ = 5150 s/mm^2^ at 7 T, and b_eff_ = 8550 s/mm^2^ at 7 T, respectively. These are all statistically significant different from one another (*p* < 0.05, Shapiro–Wilk test for normality followed by the non-parametric Kruskal–Wallis test). There is a decrease in the number of voxels where secondary fiber estimates were produced in white matter at both lower field strength and high b_eff_. Decreased populations of secondary fiber estimates at 3 T are likely due to lower diffusion CNR at 3 T (Fig. 3d). Since diffusion CNR is higher with higher b_eff_, the decrease in secondary fiber populations due to larger b_eff_ at 7 T is likely due in part to B_1_ dependent decreased SNR.

#### Primary and secondary fiber estimates

**Fig. 4:**
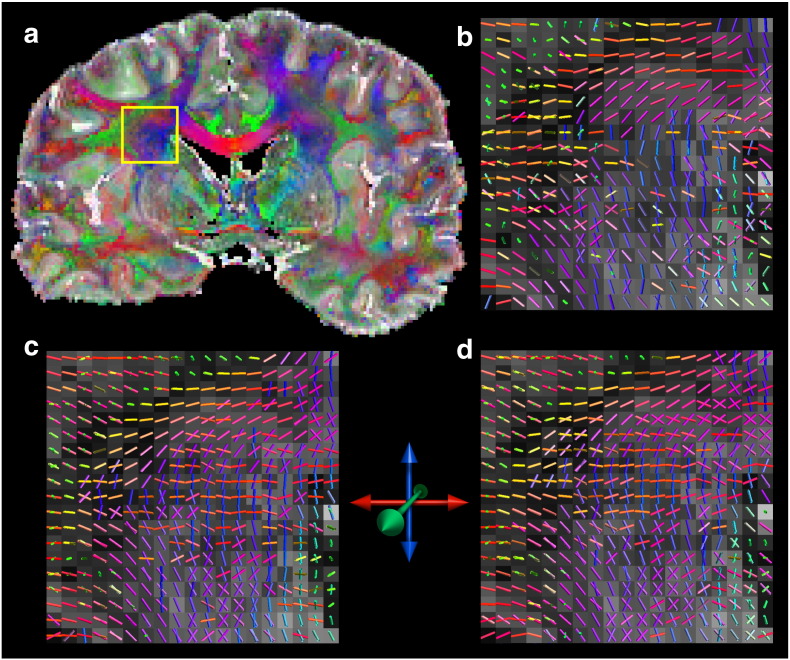
(a) Principle diffusion direction overlain on the mean diffusivity image. The yellow box indicates the regions blown up in (b–d), which show the primary and secondary fiber estimates through the centrum semiovale where both the CC/CST and CC/SLF interdigitate. Data acquire were acquired at (b) 3 T, b_eff_ = 5175 s/mm^2^, (c) 7 T, b_eff_ = 5150 s/mm^2^, and at (d) 7 T, b_eff_ = 8550 s/mm^2^. More secondary fibers are present in both 7 T data (c–d), and directional coherence appears greater between coincident voxel at higher b_eff_.

Typical results of primary and secondary fibers within the CS are shown in Fig. 4. This region of the CS is characterized by interdigitation of (i) interhemispheric fibers of the CC spanning right–left, fanning into the cortex, (ii) CST fibers spanning primarily superior–inferior, reaching the sensorimotor cortex, and (iii) SLF fibers spanning anterior–posterior. This crossing fiber structure is most clearly delineated in the 7 T data (Figs. 4b,c). Several trends underlying the pooled results in Fig. 4 can be seen: the 7 T data generally has more secondary fibers in the region of the CS; secondary fibers from the CC are clearly seen crossing both the CST and the SLF at 7 T, where very few crossings are seen at all at 3 T (Fig. 4a); finally, there is greater coherence of multi-fiber architecture across neighboring voxels in the 7 T data, particularly at high b_eff_.

### B_1_ effects on DTI measurements

Previous work on DW-SSFP for post-mortem brain data at 3 T (McNab et al., 2009, Miller et al., 2011) was able to reasonably assume homogeneous B_1_ across the sample. At 7 T, however, objects with dimensions on the order of 14 cm suffer from well-established B_1_ inhomogeneities (Collins et al., 2005, Yang et al., 2002). Unlike spin-echo based measurements, the diffusion contrast in DW-SSFP depends on the excitation flip angle, making knowledge of B_1_ important for accurate quantification.

**Fig. 5:**
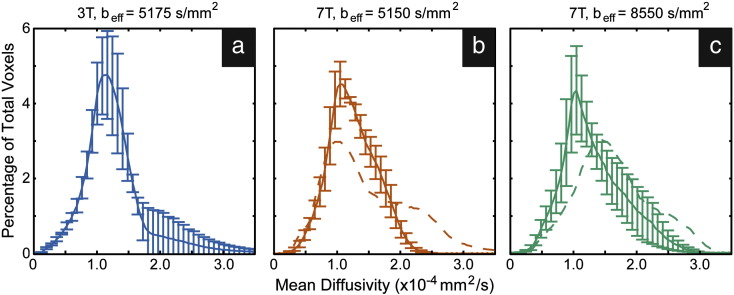
Normalized histograms of white-matter-masked mean diffusivity values (± standard error) pooled over all data for each protocol. Solid lines represent data processed with the inclusion of a B_1_ map. Dashed lines in b and c indicate 7 T data processed without B_1_ information.

Histograms of pooled white matter masked mean diffusivity (MD) results are shown in Fig. 5. Solid lines in each plot are for data processed with the inclusion of a B_1_ map; dashed lines (Figs. 5b,c), without. It is immediately evident that MD values produced from data processed without B_1_ information are quite different than data acquired at 3 T (Fig. 5a). A two-sample K–S test indicates that distributions of MD at 3 T statistically differ from both distributions at 7 T without the inclusion of B_1_ (*p* ≪ 0.0001). Moreover, the two distributions at 7 T without the inclusion of B_1_ are also significantly different from one another (*p* ≪ 0.0001). The inclusion of B_1,_ on the other hand, produces more agreement between field strength and diffusion weighting. In all but one of the same aforementioned tests between distributions (replacing 7 T distributions with those processed with B_1_), the null hypothesis that data come from the same distributions is not rejected; all distributions are the same. The exception is in the case of data acquired at 3 T compared with that acquired at 7 T with higher diffusion weighting. In this specific case, the distributions are statistically significantly different (*p* = 0.04).

Similar comparisons of the effects of changes in FA due to variations in field strength, b_eff_, and with or without B_1_ maps were made as well. Statistical comparison of distributions (two-sample K–S test, *p* > 0.2) indicated that FA distributions weren't statistically different regardless of the various permutations of variables. This indicates that computing FA from DW-SSFP data acquired of PM human brain is robust to the applied protocol variations.

**Fig. 6:**
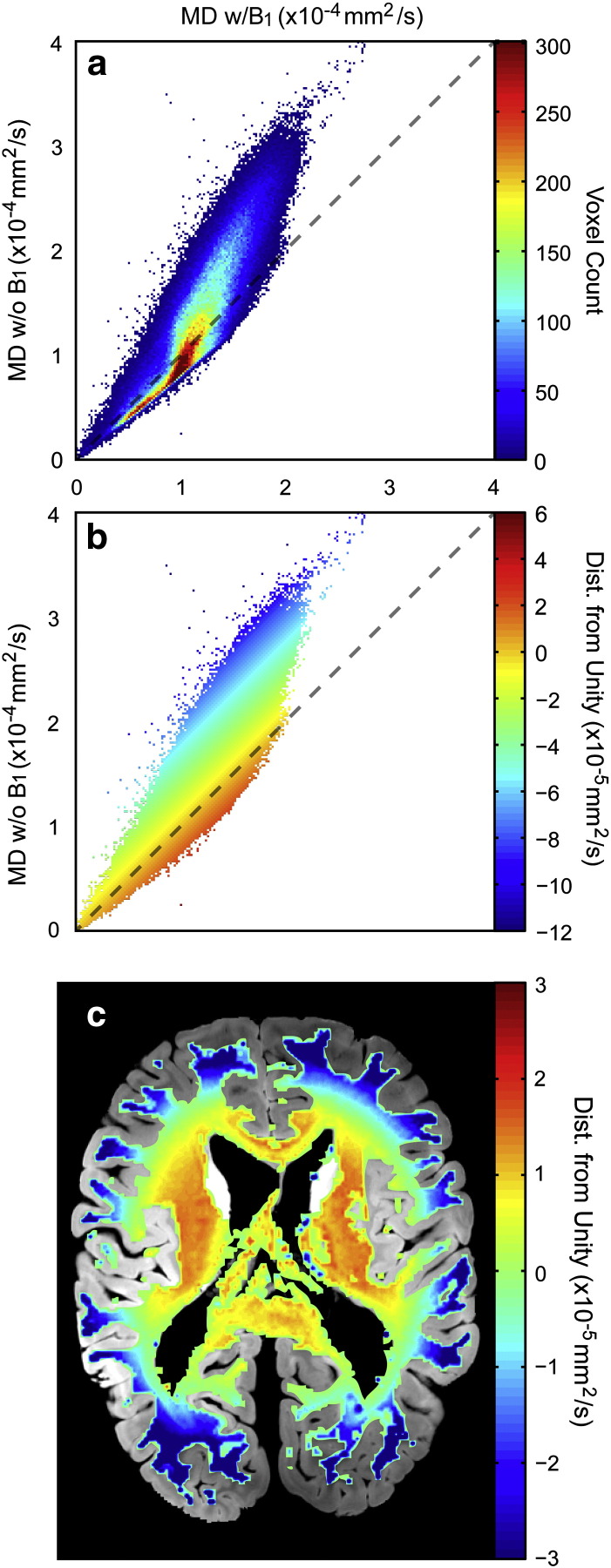
(a) Typical density plot of DW-SSFP data acquired at 7 T with b_eff_ = 8550 s/mm^2^ comparing data processed with vs. without the inclusion of a B_1_ map. Areas of highest density occur along unity (dashed line) but begin to deviate from unity as MD values become increasingly larger in data processed without a B_1_ map compared to those processed with. (b) The perpendicular distance from unity of each point in the density plot (a) was computed and color-coded. These values were mapped to structural space (c) and overlain on a structural image to give a spatial estimate of the effect that the inclusion of a B_1_ map had on the results. The distance from unity looks very similar to the B_1_ inhomogeneity expected at 7 T, where the flip angle at the center is closer to that prescribed, decreasing radially towards the cortex.

To understand the source of variability between data acquired at 7 T with and without B_1_ information 2D density plot of MD processed with and without B_1_ were produced (for the latter, we assumed a constant flip angle across the brain). Typical results are shown in Fig. 6. Deviations from unity (dashed line, Fig. 6a) show that computed MD without the inclusion of B_1_ were overall higher than those computed with B_1_ and include a much broader range of MD values. The perpendicular distance from unity for each point in the density plot was computed (Fig. 6b), color-coded, and mapped to structural space (Fig. 6c). MD estimates deviate across the brain with a spatial pattern corresponding to the B_1_ inhomogeneity pattern expected at 7 T (described more thoroughly below). The map in Fig. 6c indicates agreement for the two techniques (the orange-yellow region of the color map) in central white matter where the flip angle voltage was calibrated, with increasing discrepancies across the two analyses for peripheral areas where the B_1_ map indicates significant deviation from the prescribed flip angle.

### Tractography

**Fig. 7:**
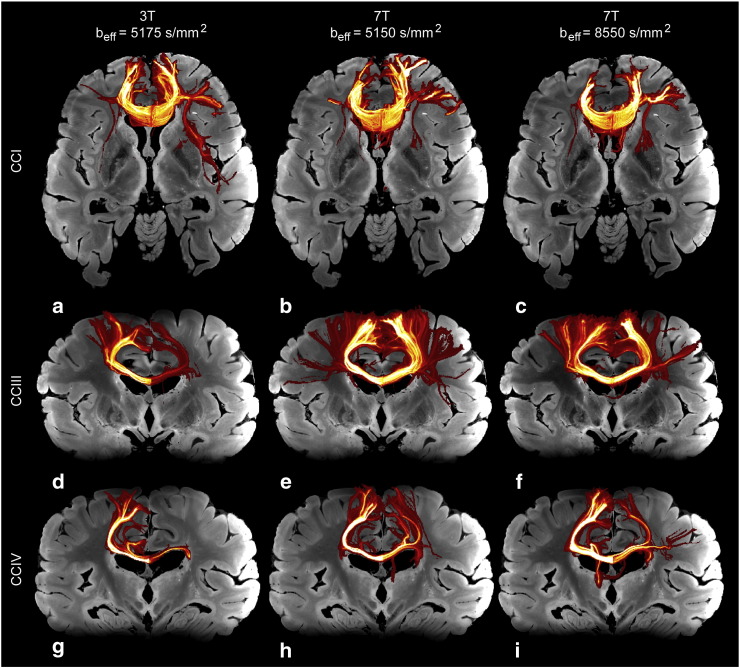
Maximum intensity projection (MIP) images of different sub-regions of the corpus callosum (CC) produced from tractography results of DW-SSFP data. MIPs are overlain on 7 T structural data. The left column is results from a typical 3 T dataset; the right, 7 T.

**Fig. 8:**
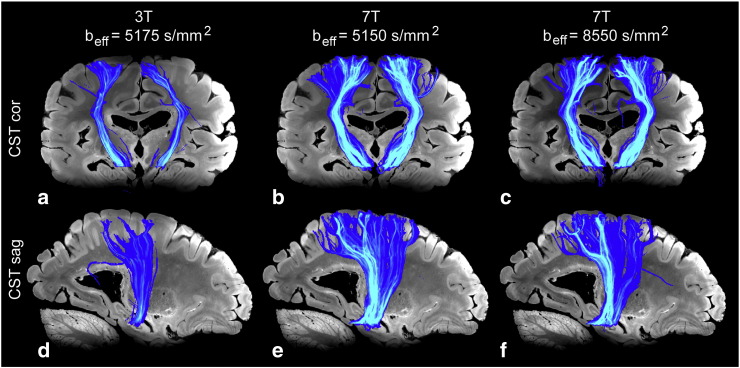
Maximum intensity projection (MIP) images of various motor projection pathways, including the CST produced from tractography results of DW-SSFP data. MIPs are overlain on 7 T structural data. The left column is results from a typical 3 T dataset; the right, 7 T. (a–b) Both the left and right CST as seen projected down the AP direction. (c–d) The right CST, as seen projected down the RL direction.

**Fig. 9:**
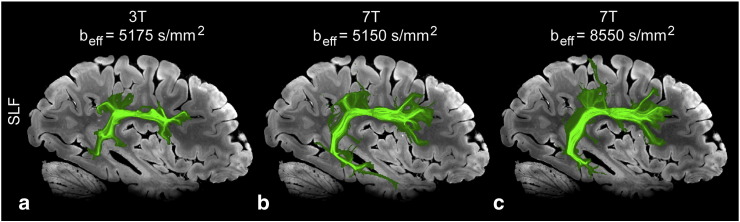
Maximum intensity projection (MIP) images of the superior longitudinal fasciculus (SLF) produced from tractography results of DW-SSFP data. MIPs are overlain on 7 T structural data. The left column is results from a typical 3 T dataset; the right, 7 T.

Fig. 7, Fig. 8, Fig. 9 demonstrate typical maximum intensity projections (MIPs) of tractography results. We targeted the three major white matter tracts that interdigitate through the CS (described above).

Figs. 7a–c show a typical MIP of the genu of the CC, projected down the axial direction. This portion of the CC has few cortical projections that cross the CS, and appears quite similar between 3 T and 7 T data. In contrast, Figs. 7d–f demonstrate MIPs of regions III and IV of the CC, which interdigitate with other tracts in the CS before reaching their cortical targets. In both cases, the 7 T results provide a superior segmentation of the known tract anatomy, whereas 3 T data tend to produce a reduced representation of the tract without the characteristic fanning of projections depicted in the 7 T data. These results suggest that, without robust estimation of the multi-fiber architecture, streamlines either terminate in the CS or mistrack into the interdigitating tracts. In either case, tracts do not reach the expected cortical projections indicated by the inclusion masks (Figs. 1b,c).

Typical MIPs of the CST projected down the coronal and sagittal directions are shown in Figs. 8a–c and d–f, respectively. 7 T data demonstrate drastic improvements in general tracking as well as an increase in streamlines reaching cortical targets. The left CST produced from 3 T data shows little tracking to the superior primary motor cortex as seen in both coronal and sagittal views. 7 T data of the same tract shows significant tracking improvements in both orientations. The improved fanning appearance of the CST at 7 T reveals the extent to which improvements in secondary fiber populations are estimated compared with 3 T data, as these estimates are necessary for these projections to be identified.

Fig. 9 demonstrates the projection of the left SLF down the axial direction. As with the previous tracts discussed, the 7 T data demonstrates a more complete tract than the 3 T results.

## Discussion

Our results clearly demonstrate an improvement in secondary fiber estimation populations in DW-SSFP data acquired at 7 T compared with 3 T using single line readout, DW steady-state free precession. These results directly translate to improved probabilistic tractography at 7 T, particularly under conditions where streamlines must cross an area known to contain interdigitating fibers. There are several findings that suggest more robust estimates of secondary fiber populations as the reason for improved tractography. First, our 7 T data clearly support improved estimation of secondary fibers, both in terms of the number of voxels where the ARD indicate a second fiber and the coherence of the estimated fibers (Fig. 4). Second, our tractography analysis specifically targeted this effect by placing inclusion masks opposite the CS from the seed such that only streamlines that successfully crossed the CS were retained (Fig. 7, Fig. 8, Fig. 9). Finally, under the one condition where fibers did not have to navigate a crossing fiber region, the genu of the CC, the differences between 3 T and 7 T data was much less striking (Figs. 7a–c), which is again consistent with fidelity of estimated crossing fibers being the driving effect behind the observed differences.

### Tractography exclusion masks

As indicated in Fig. 1, tractography was performed with exclusion masks to isolate tractography results to known anatomy. This enables isolation of true positives from large, well-established fiber bundles, thereby demonstrating improved tractography results due to better estimation of multiple fiber populations, all of which are driven by hardware improvements. However, the need for such exclusion masks reflects a well-known limitation of diffusion tractography, which is the inability to identify previously unknown tracts due to the high rate of false positives. “Tract discovery” can at present only be achieved with invasive tracers in post-mortem tissue, making direct comparison to diffusion tractography in the same tissue highly valuable.

### Tractography at 7 T using b_eff_ = 5150 s/mm^− 2^ data

Our results show that 7 T DW-SSFP data acquired with both diffusion weightings (b_eff_ = 5150 and 8550 s/mm^2^) were higher SNR and CNR than 3 T. Moreover, the angular uncertainty of secondary fiber populations was slightly improved with higher b_eff_, while the number of white matter voxels in which secondary fiber populations were estimated at all was greater in data acquired with lower b_eff_. Both of these metrics provide similarly compelling information regarding which b_eff_ provides more optimal data for subsequent tractography.

### Future work

#### Potential correction of B_1_ effects at 7 T

One source of SNR loss at higher b_eff_ is B_1_ inhomogeneity at 7 T due to the short wavelength of the RF, which is on the order of the sample itself. The high dielectric value of tissue and standing wave patterns due to the short wavelength at 7 T lead to characteristic ‘bright spots’ in images (Collins et al., 2005, Yang et al., 2002). This radial pattern of B_1_ inhomogeneity translates directly into lower flip angle in cortical regions and therefore radially dependent SNR. Additional SNR reductions associated to increasing b_eff_ can therefore lead to spatial biases in the quality of fiber reconstruction (with poor estimates in low SNR regions, such as the cortex in our data).

Signal loss due to B_1_ effects is a problem that potentially compromises all data acquired at 7 T, within a certain range of sample sizes. For example, tractography of the genu (Figs. 7a–c) is clearly compromised near the cortex at least in part due to the B_1_ dependent SNR loss in the higher b_eff_ data at 7 T (Fig. 7c) than either of the lower b_eff_ data (Figs. 7a,b). Much work has been and continues to be performed to correct for this, as it is a significant technical difficulty associated with the implementation of 7 T to a clinical setting.

One method that has been implemented with varying degrees of success is passive B_1_ shimming using bags filled with high dielectric constant material (Webb, 2011). The high dielectric serves to redistribute the RF allowing for B_1_ field shaping that is sample dependent; for simple geometric B_1_ shimming (e.g. improving RF power in regions of typical signal loss such as that found in the frontal lobe due to the sinuses) it is very promising. In instances where target regions are small, such as for specific localized anatomy or spectroscopy, local B_1_ redistribution by this method is quite effective. However, given the overall size as well as complicated geometry of the post-mortem brain surface this approach becomes less effective. Multiple efforts were made on both phantoms and brain samples to reproducibly implement this method using varying configurations of bags filled with multiple differing high dielectric constant materials, including water (Yang et al., 2006), calcium titanate, and barium titanate (Teeuwisse et al., 2012) with limited success.

Dynamic shimming using parallel transmit channels (pTx) is another technologically promising approach (Katscher and Bornert, 2006); this would overcome the geometric variability between samples and allow for precise and unique sample dependent B_1_ shimming. This represents a significant advancement in MRI in general as well as a sophisticated approach to the problem, but it requires a high level of expertise and considerable technical development.

Given that post-mortem imaging generally involves long scan times, we have begun to investigate a simpler approach using acquisition at multiple flip angles (Foxley et al., 2014). As mentioned above, the flip angle in DW-SSFP affects both the SNR and the diffusion contrast, making this an important protocol parameter to optimize. With this proposed simple approach, insufficient SNR in the cortex due to B_1_ inhomogeneity could be overcome and data could be more reliably acquired piecemeal across the whole brain.

The analysis of DW-SSFP data requires additional estimates that are not needed for DW-SE measurements. In addition to T_1_ and T_2_ maps, scanning at 7 T necessitates B_1_ maps to account for variation in flip angle, which was not required at 3 T (Miller et al., 2012). Thus, our imaging protocol requires the combination of a range of images, including not only different diffusion directions, but also T_1_, T_2_ and B_1_ maps. Importantly, this combination is facilitated by extremely low distortion due to the single-line (rather than EPI) readout. While some co-registration is required to eliminate small displacements due to hardware drift, we have developed a robust pipeline of constrained rigid-body alignments (Miller et al., 2011). As a result, these alignment and distortion problems have proven to be far less problematic than in typical in-vivo acquisitions (Jones and Cercignani, 2010).

#### Tractography validation

In order to improve the confidence and reliability of tractography results, a comparison to some kind of ‘gold standard’ is necessary, and is a longer-term goal of this research. Examples include classical dissection of frozen tissue (Ludwig and Klingler, 1956); histological techniques including polarized light imaging (Axer et al., 2000), automated axonal orientation determination (Bartsch et al., 2012), or structure tensor analysis (Budde and Annese, 2013); and passive lipid membrane transport of fluorescent tracers (Seehaus et al., 2013). Achieving a reliable correlation between tractography produced from post-mortem imaging and these inherently invasive ‘gold standard’ techniques would provide an intermediary between histology and in vivo data.

One major obstacle to achieving these goals is caused by the changes to post-mortem tissue properties (reduced T_1_, T_2_ and ADC), which makes it difficult to achieve equivalent diffusion contrast to that obtained for in-vivo diffusion imaging. For example, even with the improvements afforded by DW-SSFP and 7 T, the data presented here cannot reliably extract three fiber populations for voxels in the CS, which has been demonstrated in vivo (Wedeen et al., 2008). Improvements in diffusion contrast are the subject of on-going research. In spite of these challenges, post-mortem diffusion imaging offers opportunities to improve spatial resolution, potentially providing a link between the gold-standard techniques mentioned above, which are inherently invasive to tissue, and in-vivo imaging, which suffers from poor specificity. High-resolution data directly improves the quality of tracking, for example tracing the entirety of the cingulum bundle from a single seed mask (Miller et al., 2012) where previous work has required a multi-step seeding approach (Wakana et al., 2004), as well as highly localized features of fiber architecture, for example cortical anisotropy (McNab et al., 2009, Miller et al., 2012).

## Conclusion

Significant improvements in overall data quality and subsequent tractography indicate that DW-SSFP is well suited for acquiring diffusion data in post-mortem brain. Further, DW-SSFP data acquired on our 7 T scanner confers SNR and CNR benefits over our previous 3 T hardware. These likely come from a range of hardware improvements (B_0_, receive channels, gradient strength). Ideally, higher b_eff_ would be used than reported previously to overcome ADC reductions due to death and fixation, such that post-mortem imaging would be directly comparable to in vivo imaging. Our comparisons across b-value are equivocal, suggesting the need for further work. Data acquired at higher field strength demonstrated improved voxel-wise crossing fiber estimates, and accompanying improvements in tractography through crossing fiber regions (specifically through the CS). Finally, we investigated the effects that B_1_ inhomogeneity at 7 T has on tensor estimate results. We show that simply including a B_1_ map in the analysis brings estimated diffusion metrics into line with 3 T data; however, B_1_ inhomogeneity also creates variable SNR and contrast in DW-SSFP, which will be another area of future research.

## Abbreviations

DTI: diffusion tensor imaging
DW: diffusion-weighted
MRI: magnetic resonance imaging
SNR: signal-to-noise ratio
PMI: post-mortem interval
SI: scan interval
FA: fractional anisotropy
ADC: apparent diffusion coefficient
DW-SE: diffusion-weighted spin echo
DW-SSFP: diffusion-weighted steady-state free precession
b_eff_: effective b-value
CS: centrum semiovale
CC: corpus callosum
CST: corticospinal tract and other related motor cortex projections
SLF: superior longitudinal fasciculus
MIPS: maximum intensity projection images
ARD: automatic relevance determination

## Appendix A Supplementary

Supplementary Fig. 1 Histogram of SNR at 7 T as a function of the fraction of the applied nominal flip angle due to B_1_ inhomogeneities. ROIs were defined from white matter masked b = 0 s/mm^2^ data and thresholded by the range of normalized B_1_ map values, as indicated by the x-axis. SNR clearly increases as the fraction of the nominal applied flip angle approaches 1 (the SNR efficiency optimized flip angle).

Supplementary Fig. 2 Tractography results of the optic radiation. Seed masks and inclusion masks were defined according to Winston et al. (2011). Blue lines in axial images indicate the position of the respective coronal insets. Tracts at 7 T have a greater propensity for impinging on the visual cortex with both b_eff_ values than at 3 T. Qualitative observation demonstrates that B_1_ inhomogeneities are less dramatic in the occipital lobe than in the frontal lobe. This suggests that with sufficient SNR in the distal regions from the deep white matter (i.e. the cortex) where B_1_ inhomogeneities become a concern, tractography results at 7 T out-perform those at 3 T. The variability between the tracts at 7 T with differing b_eff_ values serves to underscore the need to continue efforts to improve the acquisition protocol, including overcoming effects of B_1_ inhomogeneity as well determining the optimal b_eff_ value.

## Footnotes

1 Fomblin (or a similar liquid) is an ideally suited material for tissue submersion as it is a biologically and chemically inert, proton-free perfluoropolyether oil that contributes no signal to the imaging experiment, while simultaneously providing a susceptibility-matched surrounding environment to prevent related artifacts.
