# Supplementary figures and images for: Improving diffusion-weighted imaging of post-mortem human brains: SSFP at 7 T
Source: Neuroimage. 2014 Nov 15;102:579–89. doi: 10.1016/j.neuroimage.2014.08.014 (PMC4229505; doi:10.1016/j.neuroimage.2014.08.014)

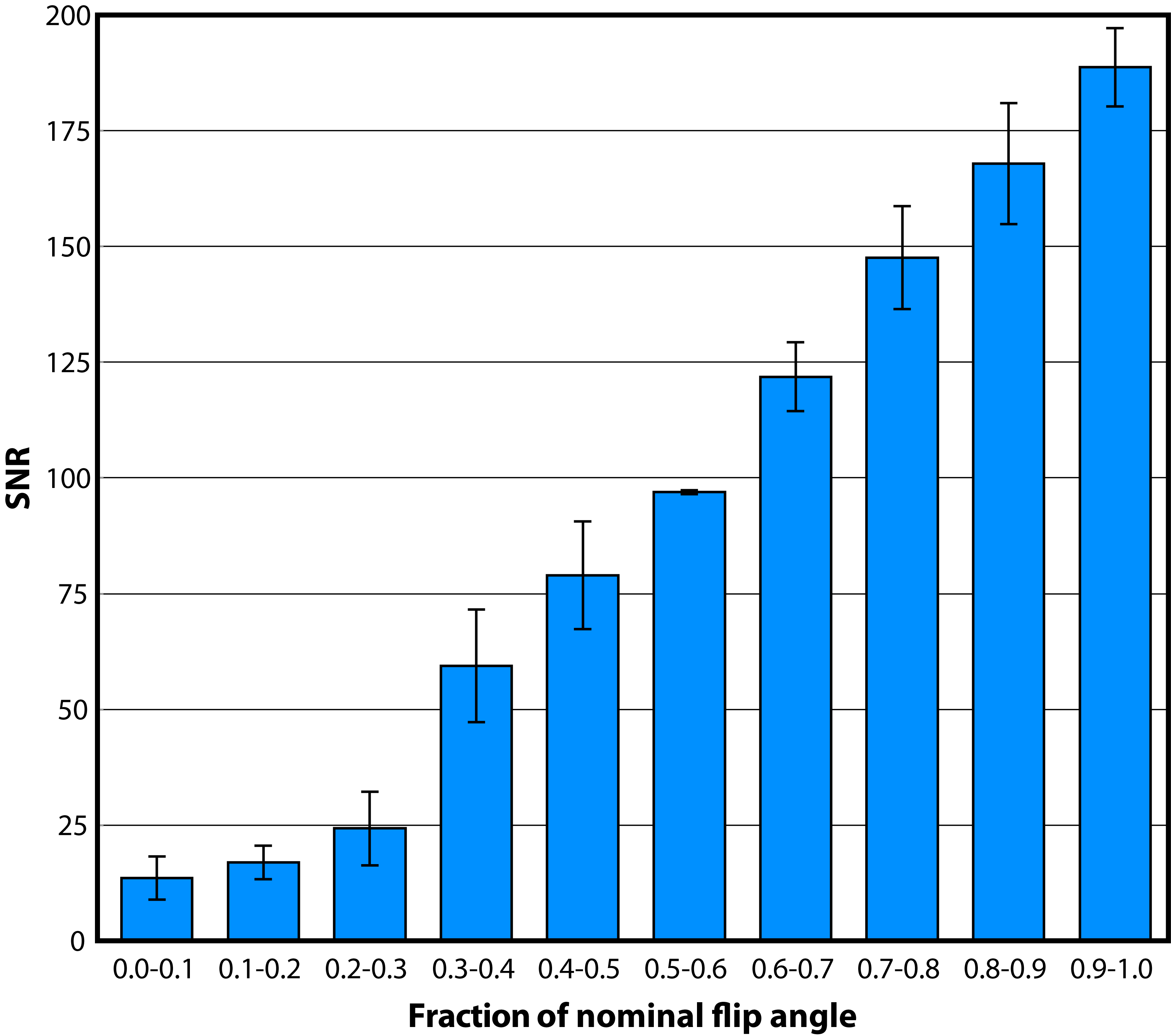

Supplement: Supplementary Fig. 1 — Histogram of SNR at 7 T as a function of the fraction of the applied nominal flip angle due to B1 inhomogeneities. ROIs were defined from white matter masked b = 0 s/mm2 data and thresholded by the range of normalized B1 map values, as indicated by the x-axis. SNR clearly increases as the fraction of the nominal applied flip angle approaches 1 (the SNR efficiency optimized flip angle). [file mmc1.zip › nim11583-mmc1.tif]

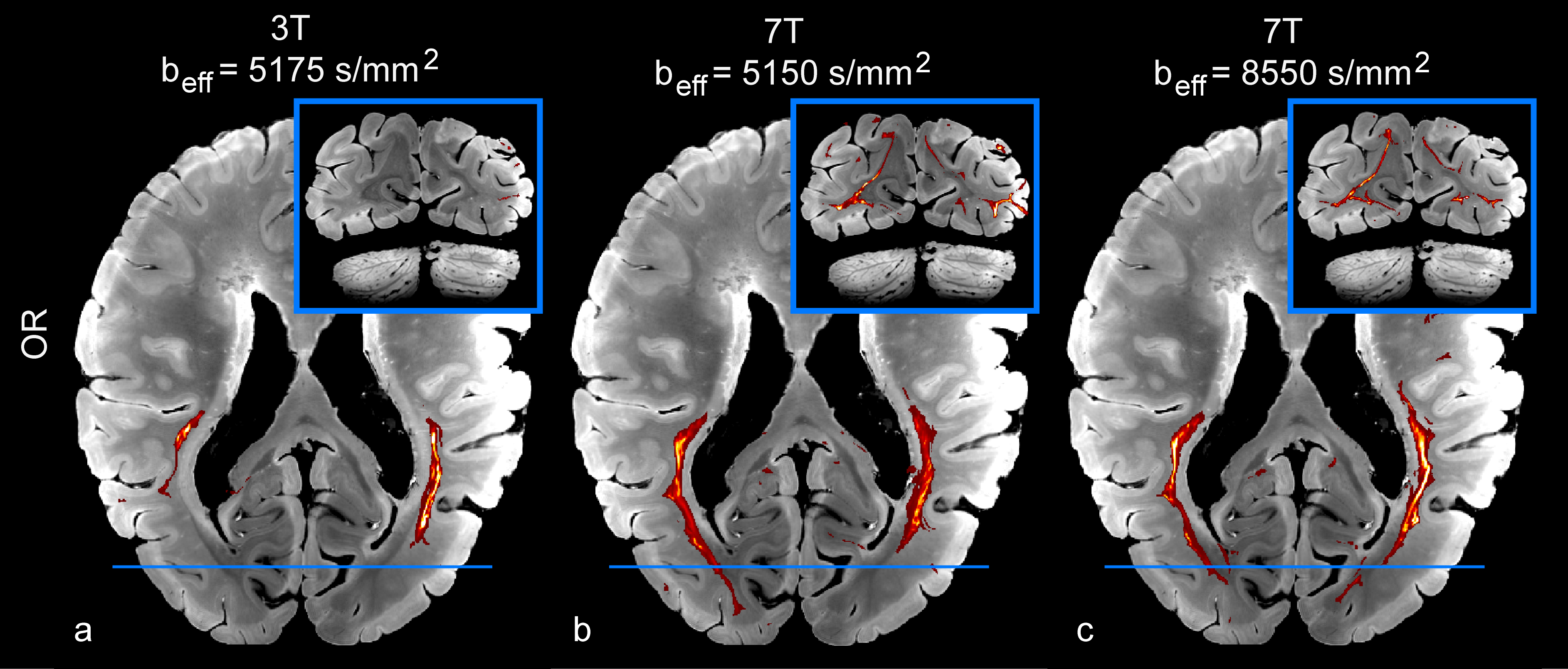

Supplement: Supplementary Fig. 2 — Tractography results of the optic radiation. Seed masks and inclusion masks were defined according to Winston et al. (2011). Blue lines in axial images indicate the position of the respective coronal insets. Tracts at 7 T have a greater propensity for impinging on the visual cortex with both beff values than at 3 T. Qualitative observation demonstrates that B1 inhomogeneities are less dramatic in the occipital lobe than in the frontal lobe. This suggests that with sufficient SNR in the distal regions from the deep white matter (i.e. the cortex) where B1 inhomogeneities become a concern, tractography results at 7 T out-perform those at 3 T. The variability between the tracts at 7 T with differing beff values serves to underscore the need to continue efforts to improve the acquisition protocol, including overcoming effects of B1 inhomogeneity as well determining the optimal beff value. [file mmc2.zip › nim11583-mmc2.tif]
